# Autoimmune hypothyroidism and intermittent ovarian failure - Case Report

**DOI:** 10.5935/1518-0557.20190015

**Published:** 2019

**Authors:** Ana K. Bartmann, Leticia D.F. Silveira, Liliane F.I. Silva, Flavia S.S. Formolo, Juliana P. do Amaral, Heloisa M Serra, Luciana C. S. Frolich

**Affiliations:** 1 Human Reproduction Center of the Ana Bartmann Clinic, Ribeirão Preto, SP, Brazil; 2 Medical School, University of Ribeirão Preto (UNAERP), SP, Brazil; 3 Biotechnology Department, University of Ribeirão Preto (UNAERP), SP, Brazil

**Keywords:** ovarian failure, premature ovarian failure, autoimmunity

## Abstract

Case presentation: a 35 year-old physical educator sought gynecological care for secondary amenorrhea and infertility. She denied the occurrence of similar problems in her family and referred to hypothyroidism as her only comorbidity, for which she was on levothyroxine 88µg daily. She was tested for beta-HCG, prolactin and TSH levels. She was negative for beta-HCG, and had prolactin and TSH levels of 19ng/ml and 2.04 mIU/ml, respectively. Her progesterone test was negative. The combined test (estradiol + norethisterone acetate) was positive, excluding the possibility of an anatomical cause. One month later, her blood tests were as follows: FSH 100mIU/ml, TSH 1.54mIU/ml, free T4 1.22ng/dl, and anti-TPO 261U/ml. Her FSH level was above 100 and she was diagnosed with premature ovarian failure. Reproductive treatment with donor eggs was proposed as an option. Karyotyping and a test for fragile X syndrome were ordered. A few months later the patient came to our clinic saying she was having menstrual cycles. Blood tests were as follows: FSH 9.2mIU/ml; TSH 2.21mIU/ml; and anti-TPO 14U/ml. Transvaginal ultrasound showed a normal uterus with a thin endometrium and atrophic ovaries. After two years of irregular menstrual cycles, she became amenorrheic again. She chose not to undergo assisted reproduction. This paper discusses the diagnosis of premature ovarian failure in light of current protocols and the association of this condition with diseases such as Hashimoto's thyroiditis, and looks into the difficulty of performing differential diagnosis against Savage syndrome and of offering reproductive counseling especially in cases where the menstrual cycle returns.

## CASE PRESENTATION

A 35-year-old physical educator sought gynecological care for secondary amenorrhea and infertility. She denied the occurrence of similar problems in her family and referred to hypothyroidism as her only comorbidity, for which she was on levothyroxine 88 µg daily. She denied drug or alcohol abuse.

She was initially tested for beta-HCG, prolactin and TSH levels. Her beta-HCG test was negative, and her prolactin and TSH levels were 19ng/ml and 2.04mIU/ml, respectively. Her progesterone test was negative. The combined test (estradiol + norethisterone acetate) was positive, excluding the possibility of an anatomical cause. The patient had no history of uterine surgery. One month after the combined test, the patient performed new blood tests, which read as follows: FSH 100mIU/ml, TSH 1.54mIU/ml, free T4 1.22ng/dl, and anti-TPO 261U/ml.

She was diagnosed with premature ovarian failure based on her extremely high FSH level (above 100) and reproductive treatment with donor eggs was proposed as an option. Karyotyping and a fragile X syndrome test were requested. The patient was referred for evaluation with a clinical geneticist, although both tests were normal.

A few months later, however, the patient came back to the clinic saying she was having menstrual cycles. New tests were performed and read as follows: FSH 9.2mIU/ml; TSH 2.21mIU/ml; and anti-TPO 14U/ml. Transvaginal ultrasound examination revealed a uterus of normal volume and size, with a thin endometrium (3mm) and atrophic ovaries. After 18 months of irregular menstrual cycles, the patient became amenorrheic again. She chose not to undergo assisted reproduction.

This paper discusses the diagnosis of premature ovarian failure in light of current protocols and the association of this condition with diseases such as Hashimoto's thyroiditis. It also looks into the troubles of performing the differential diagnosis against Savage Syndrome and of offering reproductive counseling especially in cases where the menstrual cycle returns.

## DISCUSSION

 Premature ovarian failure (POF) is defined as the loss of ovarian follicular activity at an age fewer than two standard deviations from the mean age of menopause in the population, i.e., before 40 years of age (Bartmann, 2018; Assumpção, 2014), concomitantly with high levels of FSH (Nácul, 2018). For diagnostic purposes, FSH levels >30mIU/mL are required in two tests performed 30 days apart from each other or FSH >100mIU/mL in only one test (Bartmann, 2018). These cases do not include surgically induced menopause. POF affects about 1% of women before the age of 40 and 0.1% before the age of 30 (Nácul, 2018).

Although most patients do not present prior menstrual changes suggestive of future gonadal failure, physicians must investigate the patient's and her relatives’ menstrual history and look into conditions that might cause it. Among them are Turner’s mosaicism, exposure to toxins (e.g.: pesticides, irradiation, chemotherapy), surgery, ovarian torsion, autoimmune diseases (e.g.: Hashimoto's thyroiditis and Addison's disease), and fragile X syndrome (Bartmann, 2018; Nácul, 2018).

The pathophysiology of the disease can be divided into primary (genetic and autoimmune causes) and secondary (Assumpção, 2014). In primary POF, genetic aberrations involving the X chromosome (monosomies, trisomies, translocations, deletions) or autosomes are present (Assumpção, 2014). There is a decrease in the pool of primordial follicles and an increase in atresia due to apoptosis or follicular maturation failure (Nácul, 2018; Vujović *et al*., 2012). Autoimmune ovarian failure, also a primary cause of POF, is caused by alteration of T cell subsets, T cell-mediated injury, increased production of B cell autoantibodies and decreased NK cells, as well as their activity (Nácul, 2018; Assumpção, 2014; Vujović *et al*., 2012). POF is considered secondary when it occurs due to infection, bilateral oophorectomy, chemotherapy, or radiotherapy (Assumpção, 2014; Pardini *et al*., 2006; Pardini, 2012).

The differential diagnosis against Savage Syndrome, characterized by a defect in FSH receptors, is quite difficult (Bartmann, 2018). In this case, the receptor may be genetically defective or have undergone secondary alteration due to conditions such as autoimmune diseases, but there is no obliteration of germ cells as in POF. Prognosis is quite poor, but there are reports of live births following in vitro oocyte maturation (IVM) (Li *et al*., 2016).

In complementary investigation, once gestation has been ruled out, tests for prolactin, TSH, FSH, and estradiol are ordered (Nácul, 2018). High levels of FSH (>30 mIU/mL) combined with low estradiol levels and clinical complaints of hypoestrogenism are indicative of hypergonadotropic hypogonadism (Nácul, 2018). Karyotyping is warranted in all cases of POF, regardless of the age of the woman (Nácul, 2018; American College of Obstetricians and Gynecologists Committee on Genetics, 2006). Tests for autoimmune endocrine diseases are also indicated, since they often accompany autoimmune oophoritis (Nácul, 2018; Pardini *et al*., 2006; Pardini, 2012). If there is doubt about the patient's reproductive capacity, anticoagulant follicles can be counted with the aid of transvaginal ultrasound examination and AMH (anti-Müllerian hormone). However, patients must be prepared to receive news that they have a poor reproductive prognosis regardless of the etiology of POF (Romão & Navarro, 2013).

The numerous etiologies and variable progression of POF confound physicians and often leave patients feeling insecure (Vilodre *et al*., 2007). It is still challenging to define whether POF is a permanent or transient condition (Simões, 2015; Nunes, 2011). Temporary involvement is characteristic of autoimmune conditions, and it does not necessarily mean the end of the patient’s ovarian reserve (Lima & Lourenço, 2016; Simões, 2015; Nunes, 2011). Even in physiological conditions, most women go through significant confusion when transitioning to menopause and suffer with various clinical conditions (de Souza & Araújo, 2015).

By definition, POF is a permanent condition and requires an assertive approach: investigation of genetic diseases (Turner’s mosaicism, Fragile X syndrome), use of donor eggs when patients wish to procreate, and hormone therapy (Pardini *et al*., 2006; Pardini, 2012). In cases of temporary ovarian failure, transient oophoritis due to autoimmune disease is a highly likely cause (Pardini *et al*., 2006; Vilodre *et al*., 2007). In these cases the indication is to harvest oocytes as soon as possible, before the obliteration of germ cells precludes in vitro fertilization (Li *et al*., 2016; Arici *et al*., 2002).

One might discuss the adequacy of the name given to this condition, since the term entails an erroneous sense of permanence. Ovulation and pregnancy may occur at a later time, sometimes years after diagnosis (Pardini, 2012; Simões, 2015).

Intermittent ovarian activity and gonadotropin resistance translate into cases of spontaneous conception (Assumpção, 2014). In cases of Savage Syndrome, 5-10% of the patients achieve pregnancy after being diagnosed with the condition (Shelling, 2010; Pardini *et al*., 2006; Panay & Kalu, 2009).

It is likely that autoimmune diseases might damage initially the FSH receptors and secondarily the germ cells (Simões, 2015). Thus, ovarian insufficiency due to autoimmunity might ultimately be a representation of a deterioration of oophoritis (Lima & Lourenço, 2016; Simões, 2015). To confirm this hypothesis, further experimental studies with animal models of ovarian aggression are required.

Women diagnosed with POF go through a difficult emotional period as unforeseen infertility might ruin their life plans (Rafique *et al*., 2012; Liao *et al*., 2000). Possible outcomes include increased social anxiety, low self-esteem, and depression (Schmidt *et al*., 2006; van der Stege *et al*., 2008).

## FINAL COMMENTS

The diagnosis of premature ovarian failure has challenged physicians on account of the extensive testing required and the variable course of the disease.

As shown in this case, high FSH values (>100mIU/mL), a characteristic finding in ovarian failure, may not represent a definitive process of loss of ovarian function. The term "ovarian failure" should be abandoned in exchange for "ovarian insufficiency". If the menstrual cycle does not return, it might then be reasonable to say retrospectively that ovarian failure has become definitive.
